# Boundary and vulnerability estimation of the internal borderzone using ischemic stroke lesion mapping

**DOI:** 10.1038/s41598-020-58480-y

**Published:** 2020-02-03

**Authors:** Sylvain Grange, Rémi Grange, Pierre Garnier, Jérôme Varvat, Doina Marinescu, Fabrice-Guy Barral, Claire Boutet, Fabien C. Schneider

**Affiliations:** 1 Department of Radiology, University Hospital of Saint Etienne, Saint-Priest-en-Jarez, France; 2 Stroke Unit, University Hospital of Saint Etienne, Saint-Priest-en-Jarez, France; 3 TAPE EA7423, University of Saint Etienne, Saint-Priest-en-Jarez, France

**Keywords:** Predictive markers, Stroke

## Abstract

Distinction between deep and superficial middle cerebral artery (MCA) territories and their junctional vascular area (the internal borderzone or IBZ) constitutes a predictor of stroke patient outcome. However, the IBZ boundaries are not well-defined because of substantial anatomical variance. Here, we built a statistical estimate of the IBZ and tested its vulnerability to ischemia using an independent sample. First, we used delineated lesions of 122 patients suffering of chronic ischemic stroke grouped in deep, superficial and territorial topographies and statistical comparisons to generate a probabilistic estimate of the IBZ. The IBZ extended from the insular cortex to the internal capsule and the anterior part of the caudate nucleus head. The IBZ showed the highest lesion frequencies (~30% on average across IBZ voxels) in our chronic stroke patients but also in an independent sample of 87 acute patients. Additionally, the most important apparent diffusion coefficient reductions (−6%), which reflect stroke severity, were situated within our IBZ estimate. The IBZ was most severely injured in case of a territorial infarction. Then, our results are in favour of an increased IBZ vulnerability to ischemia. Moreover, our probabilistic estimates of deep, superficial and IBZ regions can help the everyday spatial classification of lesions.

## Introduction

Identifying factors that can predict the clinical outcome of the patients is a major concern for acute stroke management. This is of particular importance in the context of reperfusion therapy. Diffusion weighted imaging (DWI) has driven considerable interest as a biomarker of patient functional outcome. Indeed, larger DWI lesion volumes are associated with worse outcome^1,2^. Besides ischemic volume and diffusion-perfusion mismatching, early neurological predictors are limited. Nevertheless, the spatial arrangement of injuries identified on DWI can be a surrogate as it is correlated with the pathogenic mechanisms of stroke and patient outcome^3,4^; after intravenous thrombolysis^5^ or not^4,6^. Indeed, infarct location within the middle cerebral artery (MCA) territory reflects stroke severity^7,8^ and may be more influential than lesion volume^9^. Consequently, the lesion topography can motivate diverse therapeutic strategies either at the acute^5^, sub-acute or chronic stages of the illness. Moreover, working with spatial lesion pattern has several practical advantages in the emergency setup compared to volume analysis which is more complex and time consuming. MCA infarctions are commonly categorized according to the territories of the deep and the superficial perforating arteries. Additionally, it has been suggested to distinguish stroke situated in the borderzone between these two vascular fields, that is the Internal Borderzone or IBZ, from other infarct types^10,11^. Pure IBZ watershed infarcts are relatively rare^3^ and underlying physiopathological mechanisms appear to be different from other strokes^6,12^. Because of their distal situation, IBZ infarcts are likely to reflect an area of high hemodynamic susceptibility, more prone to ischemia than cortical regions^13^. Moreover, the size of IBZ lesions correlates with neurological worsening^14^, especially in patients with MCA stenosis^15^. Since the IBZ appears to be more sensitive to ischemia, we hypothesize that this region would be more frequently injured than other brain areas. Its identification on a day to day basis may help the therapeutic decision^16^. Nevertheless, the large variabilities of both the vascular anatomy^17^ and the supplied territories^18^ confuse the interpretation of the lesion pattern seen on acute images. In many cases, the distinction between the IBZ, the superficial and the deep MCA territories is not straightforward. In this work, we used a probabilistic methodology to identify the spatial distribution of the IBZ. Moreover, its susceptibility to ischemia was further tested using an independent sample of acute patients and Diffusion-weighted imaging (DWI). Whether the most frequent and the most severe (in terms of Apparent Diffusion Coefficient, ADC, reduction) injuries would be observed in the IBZ then we would conclude to an increased vulnerability of this brain region.

## Results

Among 784 patients meeting the inclusion criteria, 560 lesions appeared in the MCA territory. A follow-up MR examination could not be retrieved for 232 patients. 119 were excluded because of poor image quality, recurrent stroke, or hemorrhagic transformation. Overall, the data of 209 patients were analysed. The internal borderzone (IBZ) was estimated from 122 patients and its vulnerability to ischemia was tested on an independent sample of 87 patients.

### IBZ estimation

**Figure 1:**
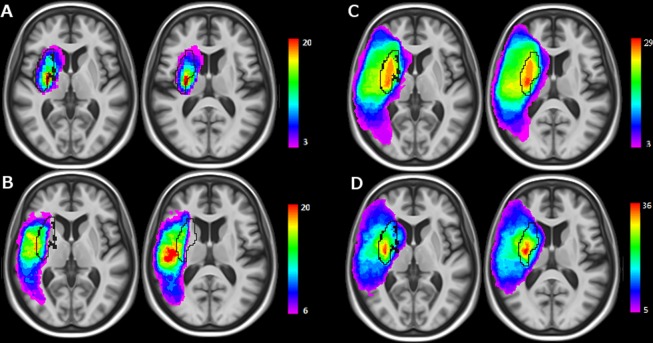
Lesion overlay plots for deep (**A**), superficial (**B**) and territorial (**C**) lesions of the sample used for the IBZ estimation. The lesion plot of the whole sample is displayed in (**D**). Warmer colors indicate increasing number of overlapping lesions. Black lines show the IBZ outer contours estimated from relation (3).

A summary of demographic, clinical and imaging results of the chronic patient sample is given in Table 1 (a more complete version is available in the Supplementary Table S1). 56 examinations were performed using scanner 1 (1T), 46 using scanner 3 (1.5T) and 21 with scanner 2 (3T). Every patient had a visible infarct on FLAIR images. 84 lesions were located in the right hemisphere. Infarcts were distributed either in the superficial (55) or the deep (38) territories and 29 were territorial. The spatial occurrence of the lesions is shown in the lesion overlay plots of Fig. 1 (a more detailed version is available in the Supplementary Fig. S1). Highest frequency of injuries was concentrated in the striato-capsular region and the insula for the deep (Fig. 1A, injured in more than 20 out of 38 individuals) and territorial (Fig. 1C, lesioned in all patients) groups. S lesions were more spatially distributed (Fig. 1B) with a maximum frequency positioned in the insula and peri-rolandic areas (about 20/55 individuals).

**Table 1:** Demographic, clinical and imaging results of chronic and acute stroke patients.

	Chronic lesions				Acute lesions			
	Deep	Superficial	Territorial	All	Deep	Superficial	Territorial	All
Number of patients	38	55	29	122	20	51	16	87
Female gender	29%	29%	45%	33%	50%	53%	63%	54%
Age (years)	64 ± 13	69 ± 12	66 ± 14	67 ± 13	67 ± 16	70 ± 14	74 ± 13	70 ± 15
NIHSS (t0)	6.6 ± 4.9	7.1 ± 7.1	15.6 ± 6.3	9.0 ± 7.3	8.4 ± 8.7	5.9 ± 5.9	16.0 ± 6.3	8.3 ± 7.7
Delay to MRI (months/hours)	2.0 ± 3.3	3.0 ± 3.3	3.9 ± 4.3	2.9 ± 3.6	4.6 ± 7.1	6.9 ± 10.0	6.3 ± 5.8	6.0 ± 8.6
Lesion Volume (cm^3^)	12.1 ± 19.2	52.6 ± 64.2	188.9 ± 126.0	72.4 ± 101.0	18.3 ± 22.7	33.6 ± 38.7	162.2 ± 127.4	53.7 ± 80.9

Average values ± standard deviations are reported. Average delay to MRI is reported in months for chronic lesions and in hours for acute strokes.

IBZ estimation from relation (3) involved the putamen, the insular cortex, the anterior part of the internal capsule, the external section of the globus pallidus, the rostral part of the corona radiata and the anterior portion of the caudate nucleus head (Fig. 1). Its volume was 21 cm^3^ which can be compared to the estimated volume of the deep (32 cm^3^) and the superficial (285 cm^3^) regions. Notably, areas of highest injury frequency of the territorial infarcts were encompassed within the IBZ illustrating the probable increased susceptibility to ischemia of this region. A mean lesion frequency was calculated across all voxels of the ROIs. In the whole sample, the voxels of the IBZ were lesioned at 32% on average (Table 2). This proportion was of 16% for both S and D regions. When considering patients with territorial stroke, the average lesion frequency was 77% within the IBZ and 41% and 40% for the D and S regions respectively.

**Table 2:** Region characteristics of chronic and acute stroke patients.

	Chronic lesions			Acute lesions			
	Volume (cm^3^)	F_all_ (%)	F_T_ (%)	F_all_ (%)	F_T_ (%)	rADC_all_	rADC_T_
IBZ	21	32	77	29	73	0.94 ± 0.12	0.81 ± 0.13
Deep	32	16	41	12	26	1.00 ± 0.12	0.96 ± 0.16
Superficial	285	16	40	15	47	0.96 ± 0.08	0.92 ± 0.09

F_all_: average lesion frequency across all region voxels and all patients, F_T_: average lesion frequency for patients with territorial stroke, rADC_all_: average rADC across all region voxels and all patients, rADC_T_: average rADC for patients with territorial stroke. Mean rADC values are reported ± standard deviations.

Moreover, gender-specific differences were estimated using Brunnel-Munzel tests. We did not find any significant difference between males and females in the IBZ, S or D topographies.

### IBZ vulnerability

**Figure 2:**
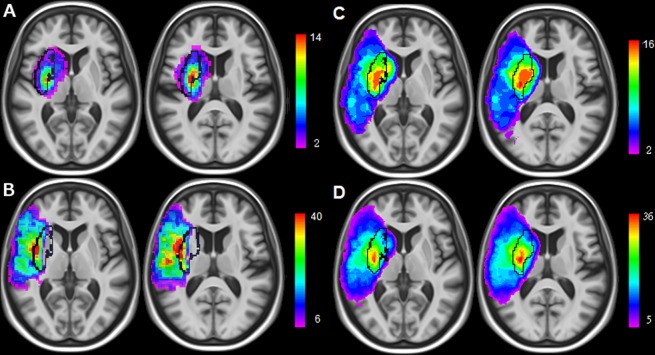
Overlay plots of the sample used to test the IBZ susceptibility to ischemia: deep (**A**), superficial (**B**), territorial lesions (**C**) and all the patients (**D**). Warmer colors indicate increasing number of overlapping lesions. Black lines show the IBZ outer contours estimated from the chronic patient sample.

**Figure 3:**
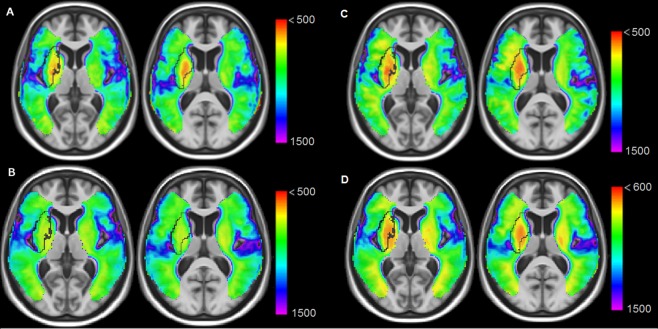
Average ADC maps for patients with deep (**A**), superficial (**B**) and territorial stroke (**C**). The whole sample is shown in (**D**). Dark lines contour the IBZ estimate (from the chronic patient sample). Warmer colors indicate more important ADC reductions (units are 10^−6^ mm^2^/s). For display purposes, regions with normal ADC values are not displayed (>0.0015 mm^2^/s). Stronger ADC decreases are within the IBZ, especially for patients with territorial stroke (**C**).

A summary of main demographic, clinical and imaging results of the acute stroke patients is given in Table 1 (and Supplementary Table S2). DW images showed an ischemic infarct for every patient. 45 lesions were situated in the right hemisphere, 16 were territorial, 51 superficial and 20 deep. 7 patients were imaged with scanner 1 (3T), 13 with scanner 2 (3T) and 67 using scanner 3 (1.5T). Overlay plots (Fig. 2, Supplementary Fig. S3) were comparable to the ones of the chronic lesion sample. Lesions were most frequently detected within the IBZ estimate of the chronic patient sample. Of note, all patients with a territorial stroke showed an IBZ injury. The average lesion frequency was of 29% across all voxels of the IBZ in the whole sample whereas 12% and 15% for D and S regions. For territorial lesions, the average lesion frequency of the IBZ was 73% while 26% and 47% in S and D regions (Table 2). Beside frequency of injuries, Fig. 3 (and Supplementary Fig. S4) illustrates averages of individual ADC maps. The IBZ showed the most severe ADC reductions. Average rADC values across all patients (±standard deviations) were 0.96 ± 0.08, 0.94 ± 0.12 and 1.00 ± 0.12 for S, IBZ and D regions respectively (P < 0.05 paired t-tests). The rADC was particularly reduced in the IBZ (0.81 ± 0.13) for patients with territorial lesions compared to S and D areas (0.92 ± 0.09 and 0.96 ± 0.14, P < 0.001). Taken together, our DWI results revealed more frequent and more severe stroke lesions within the IBZ at the acute stage of the illness.

## Discussion

Our study provides a probabilistic estimate of the IBZ and we report more frequent and more severe stroke lesions in this region. Where the watershed/borderzone regions do exactly lie between the different vascular territories is a longstanding issue^18^. To the best of our knowledge, our results are the first probabilistic estimate of the internal borderzone (IBZ) topography. Figure 1 illustrates the cerebral structures concerned by this MCA junctional territory: the putamen, the insular cortex, the anterior part of the internal capsule, the external section of the globus pallidus, the rostral part of the corona radiata and the anterior portion of the caudate nucleus head. These regions sustain many essential brain functions which contribute to critical symptomatology of acute stroke patients. They appear to be more frequently injured in the literature^4,8,9,13,19–24^ than any other brain areas. Several studies have connected the insula to stroke severity. Strokes involving of the insula are the largest^23^, signal MCA occlusion^21^ and are associated with poor clinical outcome^23,25^. In a multivariate analysis, an infarction of more 50% of the insular ribbon predicts poor outcome independently of DWI lesion volume and NIHSS^26^ (National Institutes of Health Stroke Score). Voxel-based lesion symptom mapping approaches not only linked the insula^8,23,27^ to negative functional outcome but also the corona radiata^8^ and the caudate nucleus^27^. The IBZ constitutes the vascular field irrigating these regions and they are encompassed in our IBZ estimate. Moreover, we demonstrate an increased IBZ vulnerability in two independent samples both with higher lesion frequencies (at the early, Fig. 2, and late, Fig. 1, stages) and with stronger ADC reductions (Fig. 3). We propose that our results and the ones of previous reports reflect the particular susceptibility of the IBZ to ischemia which may trigger large infarction and poor patient outcome.

Infarct volume is a biomarker of stroke severity. However, to date, lesion volumes have shown only moderate correlation with long-term clinical outcome measures^2^. Recent studies^5,7,28,29^ demonstrated that models taking into account accurate lesion topography (in addition to lesion volume) improve stroke prediction outcome and identify patients likely to benefit most aggressive reperfusion intervention and personalized rehabilitation therapies. Indeed, when deciding whether to perform reperfusion therapy, it is important to confirm whether the lenticulostriate arteries are involved in the ischemia^16,30,31^. The distinction of deep from the IBZ territories was also shown to be important^10,11^ for these considerations. However, it can be puzzling for the radiologist to clearly differentiate the superficial, deep and IBZ territories because of the anatomic variability of these vascular fields and because of the morphologic image distortions induced by the lesions. Our report can help the classification of deep, superficial and IBZ MCA territories in the clinical setting. Indeed, the anatomical structures included in our IBZ estimate (such as the putamen, the corona radiata, …) are easily detectable and this investigation is less time-consuming than infarct volume quantification. Moreover, the DWI adaptation of the Alberta Stroke Program Early CT score (ASPECTS) has drawn controversies and correlation with DWI hypersignals appeared moderate^32,33^.

We observed the strongest ADC decreases in the IBZ of acute stroke patients (Fig. 3). This was especially the case for territorial infarcts where no collateral flow from deep or superficial systems would be possible. ADC reduction is commonly used to monitor acute stroke severity^34,35^. Larger drop of ADC values being associated with more severe stroke. Taken together that we observed both the most frequent and the most severe infarcts in the IBZ, we conclude that this region is prone to develop permanent ischemic lesion; more than any other brain areas. Our results are then in line with the hypothesis of a higher vulnerability to ischemia of the IBZ^13–15^.

**Figure 4:**
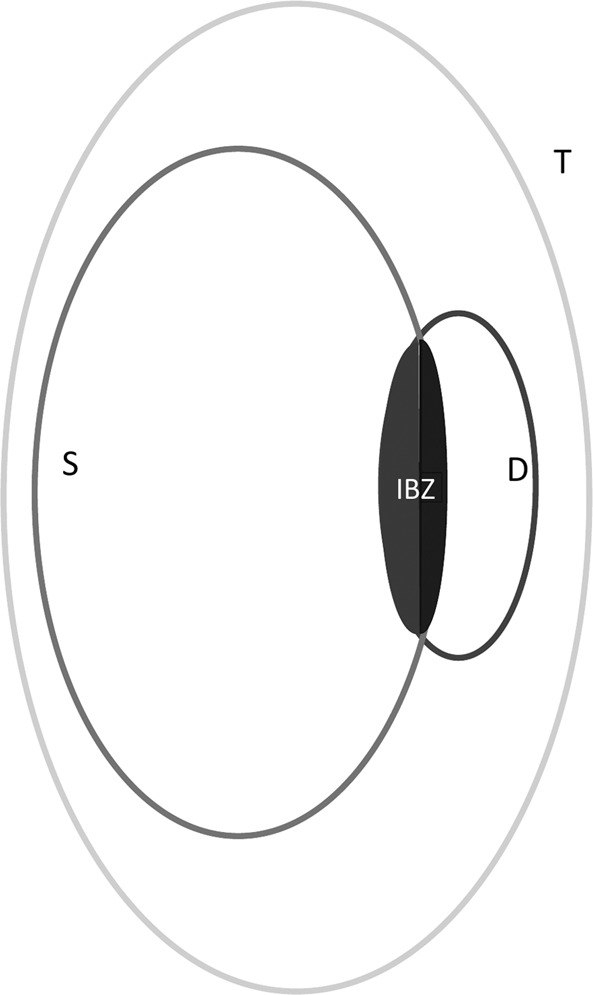
MCA sub-territories are defined according to the lesion topography: superficial (S), deep (D) or territorial (T). The border-zone between deep and superficial areas is labeled IBZ (internal borderzone).

To cartography the IBZ, a standard methodology would imply to image patients suffering of lesions exclusively within this region. However, since these infarcts are rare^3,13^ and since the vascular anatomy demonstrates considerable variability^17,36^, a large sample would then be necessary. Instead, we used an alternative methodology exploiting MCA infarcts with different topographies. This allowed the inclusion of a larger number of patients. Moreover, the work of lesions affecting the entire MCA territory also offers the chance to border the full extension of the IBZ region; capturing the spatial variance of the IBZ. Indeed, the IBZ rises from the lenticulostriate–middle cerebral artery border zone, which is supplied by the end branches of deep perforating lenticulostriate arteries and medullary penetrators from the pial–middle cerebral artery^14^. Thus a global MCA blood supply dysfunction undoubtedly encompasses the entire IBZ whereas it would be uncertain with small IBZ infarcts.

Probabilistic estimates of S, D and IBZ regions are of particular interest when considering the substantial spatial variability of the different vascular fields^24,37^. Lang *et al*.^19^ used the spatial distributions reported by van der Zwan *et al*.^37^ to study its impact on the topographical infarct characterization. Depending of the vascular variability map chosen, the detection frequency of border-zone lesions varied from 19% to 64%. In our study, we used a specific reference atlas^38^ to group the patients depending of their lesion location (deep, superficial or territorial). As a consequence of this substantial vascular variability, we cannot exclude differences whether using another vascular atlas.

A potential limitation of our study lies in the use of different MR-scanners. Indeed, a larger proportion of deep lesions were investigated using a 1.5T MR-system in our chronic sample whereas most of patients with territorial and superficial infarcts were imaged at 1T (Supplementary Table SI). Furthermore, we used several scanners for the constitution of the acute sample (3 different MR-systems, Supplementary Table SII). About 80% of the images were acquired using a 1.5T magnetic field strength and 20% with two different 3T scanners. However, the impact of the use of different MR-system either on the IBZ estimation (chronic sample) or the IBZ vulnerability testing (acute sample) is indeterminate. Of note all lesions were visible on chronic FLAIR and acute DW images and we did not find any influence of the field strength on the infarct volume of each MCA sub-territories. Another limitation of our study may be linked to the exclusion of patients with haemorrhagic or recurrent strokes because of practical analysis purposes. Consequently, our results cannot be generalised to these populations.

In this work, we provide a probabilistic estimate of the IBZ location and describe the spatial variance within common clinical subdivisions of the MCA territory. Our results support the specific vulnerability to ischemia of the IBZ. A better classification of the injured territories can increase the prediction power of models based on spatial lesion patterns. Moreover, clinical decision may also benefit from our IBZ estimate especially when evaluating the risk of a reperfusion therapy.

## Methods

The topography of the internal borderzone (IBZ) was estimated using images of patients with chronic lesions and its vulnerability to ischemia was tested using an independent sample of acute patients. This retrospective analysis of clinical data was approved by the institution’s review board (Comité Éthique Territorial Ouest Rhône-Alpes *Terre d’Éthique*, IORG0007394). The research was conducted in accordance with the principles of the Declaration of Helsinki. All the patients or their legal representatives were informed of the storage of their data in a local database and of the potential use for research purpose and provided written informed consent.

### IBZ estimation

#### Patients and clinical data

The spatial distribution of the IBZ was approximated from a population of consecutive patients with chronic stroke lesions of the MCA territory. Exclusion criteria consisted of an association with hemodynamic disturbances in posterior or anterior cerebral arteries, hemorrhagic stroke, other neurological disease and recurrent stroke between acute and chronic MR examinations. Patients with bilateral lesions were also removed from further analyses because of their low occurrence and potential confounding factors (aetiology, outcome, lesion side …). Clinical data were collected within 24 hours (t0), approximately one week (t1) and three months (t2) following symptom onsets.

#### Image acquisition

Vessel status was explored using Time of Flight Magnetic Resonance Angiography (MRA) at t0 (acute stage). Stenosis of the MCA (including occlusion) was assumed either with a reduction of more than 50% of the signal column, a signal discontinuity or with a complete signal loss. It was considered as proximal when appearing between the carotid and the M1 segment and distal in any other segments. Volumetric analysis of stroke lesions was performed at t2 (chronic stage) using the Fluid Attenuated Inversion Recovery (FLAIR) sequence. FLAIR images were interpreted by three independent experienced neuroradiologists (CB, FS, SG) blinded to clinical data. Infarct locations were categorized in three vascular distributions: deep, superficial or territorial (when involving both subdivisions) according to a reference atlas^38^. Imaging was performed using whichever one of three different scanners and the following MR protocols:

- Scanner 1: 1T Magnetom Impact Expert (Siemens, Erlangen, Germany). FLAIR parameters: TI/TR/TE 2200/9000/119 ms, FOV 230 × 230 mm, slice thickness 5 mm with an inter-slice gap of 0.5 mm, acquisition matrix 256 × 256. MRA: TR/TE 37/9.6 ms, FA 20°, FOV 250 × 250 mm, matrix 512 × 512, slice thickness 1.3 mm.
- Scanner 2: 1.5T Magnetom Aera (Siemens, Erlangen, Germany). FLAIR parameters: TI/TR/TE 2370/8000/79 ms, FOV 256 × 256 mm, slice thickness 4 mm with an inter-slice gap of 0.4 mm, acquisition matrix 256 × 256. MRA: TR/TE 25/7.15 ms, FA 20°, FOV 180 × 180 mm, matrix 512 × 512, slice thickness 0.5 mm.
- Scanner 3: 3T Magnetom Verio (Siemens, Erlangen, Germany). FLAIR parameters: TI/TR/TE 2500/9000/92 ms, FOV 240 × 217.5 mm, slice thickness 3 mm with an inter-slice gap of 0.3 mm, acquisition matrix 300 × 272. MRA: TR/TE 23/3.82 ms, FA 18°, FOV 210 × 210 mm, matrix 512 × 512, slice thickness 0.5 mm.

#### Lesion analysis

Infarcts were manually delineated on FLAIR sequences (t2) using Mricron (www.mricron.com). If necessary, images were left-right inverted so that all lesions appeared in the right hemisphere. Then, images were registered to the FLAIR template of the clinical toolbox (www.nitrc.org/projects/clinicaltbx/) which is based on healthy individuals with similar ages (mean: 65 years old) compared to characteristic stroke patients using SPM12 (www.fil.ion.ucl.ac.uk/spm/software/spm12) and lesion cost function masking^39^. This procedure was shown to be robust for normalizing brain scans with stroke lesions^40^. From normalized and binarized lesions, overlap maps were generated for the deep (D), superficial (S) and territorial (T) lesions. These maps show the frequency of injuries on a voxel-by-voxel basis. Statistical comparisons between groups were performed using Z-scores derived from the Brunner-Munzel test through the Non-Parametric Mapping formulation implemented in MRIcron. We used False Discovery Rate corrections (P ≤ 0.05) to balance for multiple testing. To investigate the minimal extension of the deep territory, a direct comparison of the number of deep lesions to superficial ones was performed (D vs. S). Since collateral flow could be involved either for D or S infarcts and thus reduced the lesion size, a larger expansion of the deep territory was estimated by comparing territorial to superficial lesions (T vs. S). No collateral flow was expected for territorial infarct and then (T vs. S) is likely to reflect the largest spatial distribution of deep infarcts. The deep territory was consequently estimated with:

1 $${{\rm{D}}}_{{\rm{est}}}=\{({\rm{D}} > {\rm{S}})\,{\rm{OR}}\,({\rm{T}} > {\rm{S}})\}$$

where D_est_ is the estimated extension of the deep territory, “>” indicates a Brunner-Munzel test to compare groups and “OR” is the logical operator.

Accordingly, the distribution of the superficial territory was determined using:

2 $${{\rm{S}}}_{{\rm{est}}}=\{({\rm{S}} > {\rm{D}})\,{\rm{OR}}\,({\rm{T}} > {\rm{D}})\}$$

Finally, the IBZ was defined as the intersection of the estimated deep and superficial territories:

3 $${\rm{IBZ}}=\{{{\rm{S}}}_{{\rm{est}}}\,{\rm{AND}}\,{{\rm{D}}}_{{\rm{est}}}\}$$

In the following, we refer to D and S regions to designate D_est_ and S_est_ with the exclusion of the IBZ voxels as illustrated in Fig. 4.

### IBZ vulnerability

#### Patients and clinical data

An independent sample of acute stroke patients was used to test for the IBZ susceptibility to ischemia. Clinical data were collected at t0 (acute stage) and at t2 (chronic stage).

#### Image acquisition

Images were acquired at t0 using either which one of three different scanners:

- Scanner 1: 1.5T Magnetom Aera (Siemens, Erlangen, Germany). DWI: TR/TE 5600/64 ms, FOV 256 × 256 mm, slice thickness 4 mm (gap 0.4 mm), acquisition matrix 160 × 160. FLAIR: TI/TR/TE 2370/8000/79 ms, FOV 256 × 256 mm, slice thickness 4 mm (gap 0.4 mm), acquisition matrix 256 × 256. MRA: TR/TE 25/7.15 ms, FA 20°, FOV 180 × 180 mm, matrix 512 × 512, slice thickness 0.5 mm.
- Scanner 2: 3T Magnetom Spectra (Siemens, Erlangen, Germany). DWI: TR/TE 7150/63 ms, FOV 256 × 256 mm, slice thickness 3 mm (gap 0.4 mm), acquisition matrix 160 × 160. FLAIR: TI/TR/TE 2500/9000/91 ms, FOV 218 × 256 mm, slice thickness 3 mm (gap 0.4 mm), acquisition matrix 232 × 256. MRA: TR/TE 23/3.82 ms, FA 18°, FOV 180 × 210 mm, matrix 652 × 768, slice thickness 0.6 mm.
- Scanner 3: 3T Magnetom Prisma (Siemens, Erlangen, Germany). DWI: TR/TE 7760/51 ms, FOV 240 × 240 mm, slice thickness 2 mm (gap 0.2 mm), acquisition matrix 160 × 160. FLAIR parameters: TI/TR/TE 2543/9600/88 ms, FOV 240 × 240 mm, slice thickness 2 mm (gap 0.2 mm), acquisition matrix 320 × 320. MRA: TR/TE 21/3.43 ms, FA 18°, FOV 180 × 200 mm, matrix 696 × 768, slice thickness 0.5 mm.

Apparent Diffusion Coefficient maps were generated from averages of three diffusion measurements in perpendicular directions at b = 1000 s/mm^2^ and a b = 0 image.

#### Lesion analysis

Volumetric analysis of stroke lesions was performed using Diffusion-Weighted Images and Mricron. Acute DW images were registered on acute FLAIR images for each patient. Then, FLAIR images were normalized to the MNI space using the FLAIR template of the clinical toolbox, SPM12 and lesion cost function masking. The same transformations were applied to the registered DW images, ADC maps and lesion segmentations. Lesion overlay and mean ADC maps were generated for the whole sample and for patients with deep, superficial and territorial infarcts and compared to the IBZ region estimated from the chronic patient sample. Relative ADC values (rADC) were generated by dividing ADC values of the affected hemisphere to mean ipsilateral mirrored values for each ROI (D, S and IBZ).

## Supplementary information

Supplementary information.

## Data Availability

The datasets generated during and/or analysed during the current study are available from the corresponding author on reasonable request.
