# Supplementary material for: Boundary and vulnerability estimation of the internal borderzone using ischemic stroke lesion mapping
Source: Sci Rep. 2020 Feb 3;10:1662. doi: 10.1038/s41598-020-58480-y (PMC6997399; doi:10.1038/s41598-020-58480-y)

# Online Supplement

**Boundary and vulnerability estimation of the internal borderzone using ischemic stroke lesion mapping**

Authors: Sylvain Grange^1^, MD, MSc, Rémi Grange^1^, MSc, Pierre Garnier^2^, MD, Jérôme Varvat^2^, MD, Doïna Marinescu^2^, MD, Fabrice-Guy Barral^1,3^, MD, Claire Boutet^1^, MD, PhD, Fabien C. Schneider^1*^, PhD.

^1^ Department of Radiology, University Hospital of Saint Etienne, France.

^2^ Stroke Unit, University Hospital of Saint Etienne, France.

^3^ TAPE EA7423, University of Saint Etienne, France.

^*^ Corresponding author: Fabien Schneider, Radiologie Centrale, CHU de Saint Etienne, 42055 Cedex 2, France. Phone : +33-477-127-529. Fax: +33-477-120-547. fabien.schneider@univ-st-etienne.fr.

**Legends of supplementary Figures**

Supplementary Figure S1: Lesion overlay plots for deep (A), superficial (B), territorial (C), and all (D) infarcts of the sample used for the IBZ estimation. Warmer colors indicate increasing number of overlapping lesions. Black lines show the IBZ outer contours. Montreal Neurological Institute coordinates of each transverse section (z axis) are given at the top of the frame.

Supplementary Figure S2: Voxel-wise statistics for deep (A) and superficial (B) MCA territories. D_est_ of relation (1) is calculated from “T>S” (A, red) and “D>S” (A, blue). Accordingly, S_est_ of relation (2) is derived from “T>D” (B, red) and “S>D” (B, blue). The color range indicates Z-scores resulting from Brunner–Munzel tests. Higher Z-scores (and color intensity) show areas associated with more chances of difference. Correction for multiple comparisons was achieved by permutation false discovery rate with a threshold of 5% causing different Z-score thresholds depending of the comparison (2.0 for “T>S”, 2.2 for “D>S”, 1.8 for “T>D”, and 1.9 for “S>D”). Dark lines contour the resultant IBZ estimation.

Supplementary Figure S3: Overlay plots of the sample used to investigate the IBZ vulnerability. Patients with deep lesions are illustrated in A, with superficial strokes in B, with territorial infarcts in C and the whole group in D. Dark lines outline the IBZ estimation obtained from the chronic patient sample. Warmer colors indicate increasing number of overlapping lesions.

Supplementary Figure S4: Average ADC maps for the 87 patients of the sample used to investigate the IBZ vulnerability. Patients with deep lesions are illustrated in A, with superficial strokes in B, with territorial infarcts in C and the whole groups in D. Dark lines contour the IBZ estimation. Warmer colors indicate more important ADC reductions (units are 10^-6^ mm^2^/s). For display purposes, regions most likely to be normal are not shown (ADC values > 0.0015 mm^2^/s).

**Supplementary Table SI**: Patient characteristics of the chronic patient sample used for IBZ estimation

|  | Deep | Superficial | Territorial | All |
| --- | --- | --- | --- | --- |
| Number of patients | 38 | 55 | 29 | 122 |
| Female gender | 29% | 29% | 45% | 33% |
| Age (years) | 64±13 | 69±12 | 66±14 | 67±13 |
| NIHSS (t0)*Ɨ | 6.6±4.9 | 7.1±7.1 | 15.6±6.3 | 9.0±7.3 |
| mRS (t1)*Ɨ |  |  |  |  |
| [0;2] | 62.9% | 57.4% | 7.2% | 47.0% |
| ]2;3] | 11.4% | 13.0% | 21.4% | 14.5% |
| ]3;6] | 25.7% | 29.6% | 71.4% | 38.5% |
| mRS (t2)*Ɨ |  |  |  |  |
| [0;2] | 75.0% | 83.0% | 45.5% | 71.1% |
| ]2;3] | 5.0% | 8.8% | 4.5% | 6.0% |
| ]3;6] | 20.0% | 11.8% | 50.0% | 22.9% |
| TOAST-I LAA | 28.9% | 21.8% | 24.1% | 21.1% |
| TOAST-II CE | 26.3% | 43.7% | 27.6% | 33.6% |
| TOAST-III SVO*# | 21.1% | 0.0% | 0.0% | 16.4% |
| TOAST-IV OT*Ɨ | 2.6% | 7.2% | 27.6% | 8.2% |
| TOAST-V UD | 21.1% | 27.3% | 20.7% | 22.2% |
| Stenosis/occlusion (acute MRA) |  |  |  |  |
| distal#Ɨ | 3% | 55% | 0% | 26% |
| proximal#* | 22% | 2% | 83% | 28% |
| Intravenous thrombolysis | 13% | 11% | 17% | 13% |
| Mean delay to MRI (months) | 2.0±3.3 | 3.0 ±3.3 | 3.9±4.3 | 2.9±3.6 |
| Scanner 1, 1T (% patients) | 16% | 55% | 69% | 46% |
| Scanner 2, 1.5T (% patients) | 66% | 29% | 14% | 37% |
| Scanner 3, 3T (% patients) | 18% | 16% | 17% | 17% |
| Lesion Volume (cm3) #Ɨ* | 12.1±19.2 | 52.6 ±64.2 | 188.9±126.0 | 72.4±101.0 |

Average values ± standard deviations are reported. Significant differences (p˂0.05) are symbolized # for deep vs. superficial MCA territories, * for deep vs. territorial and Ɨ for superficial vs. territorial. LAA: Large Artery Atherosclerosis, CE: Cardio-Embolic, SVO: Small Vessel Occlusion, OT: other, UD: undetermined. mRS: modified Ranking Scale. TOAST: Trial of ORG 10172 in Acute Stroke Treatment. The quality of two acute angiographic MR examinations was insufficient (deep) and not included.

**Supplementary Table SII**: Patient characteristics of the acute patient sample used to test IBZ vulnerability

|  | Deep | Superficial | Territorial | All |
| --- | --- | --- | --- | --- |
| Number of patients | 20 | 51 | 16 | 87 |
| Female gender | 50% | 53% | 63% | 54% |
| Age (years) | 67±16 | 70±14 | 74±13 | 70±15 |
| NIHSS (t0) | 8.4±8.7 | 5.9±5.9 | 16.0±6.3 | 8.3±7.7 |
| mRS (t2) |  |  |  |  |
| [0;2] | 50.0% | 76.5% | 25% | 60.9% |
| ]2;3] | 5.0% | 3.9% | 0.0% | 24.1% |
| ]3;6] | 45.0% | 19.6% | 75.0% | 15.0% |
| TOAST-I LAA | 20.0% | 27.5% | 43.8% | 28.7% |
| TOAST-II CE | 40.0% | 56.9% | 50.0% | 51.7% |
| TOAST-III SVO | 15.0% | 0.0% | 0.0% | 5.7% |
| TOAST-IV OT | 5.0% | 3.9% | 0.0% | 3.4% |
| TOAST-V UD | 20.0% | 11.8% | 6.2% | 10.3% |
| MRA |  |  |  |  |
| Stenosis/occlusion | 40.0% | 45.1% | 68.8% | 56% |
| Dissection | 0.0% | 3.9% | 0.0% | 2.3% |
| Mean delay to MRI (hours) | 4.6±7.1 | 6.9±10.0 | 6.3±5.8 | 6.0±8.6 |
| Scanner 1, 1,5T (% patients) | 85% | 76% | 69% | 77% |
| Scanner 2, 3T (% patients) | 5% | 18% | 19% | 15% |
| Scanner 3, 3T (% patients) | 10% | 6% | 12% | 8% |
| Lesion volume (cm3) | 18.3±22.7 | 33.6±38.7 | 162.2±127.4 | 53.7±80.9 |

Average values ± standard deviations are reported. LAA: Large Artery Atherosclerosis, CE: Cardio-Embolic, SVO: Small Vessel Occlusion, OT: other, UD: undetermined. mRS: modified Ranking Scale. TOAST: Trial of ORG 10172 in Acute Stroke Treatment.

**Supplementary Figure S1**


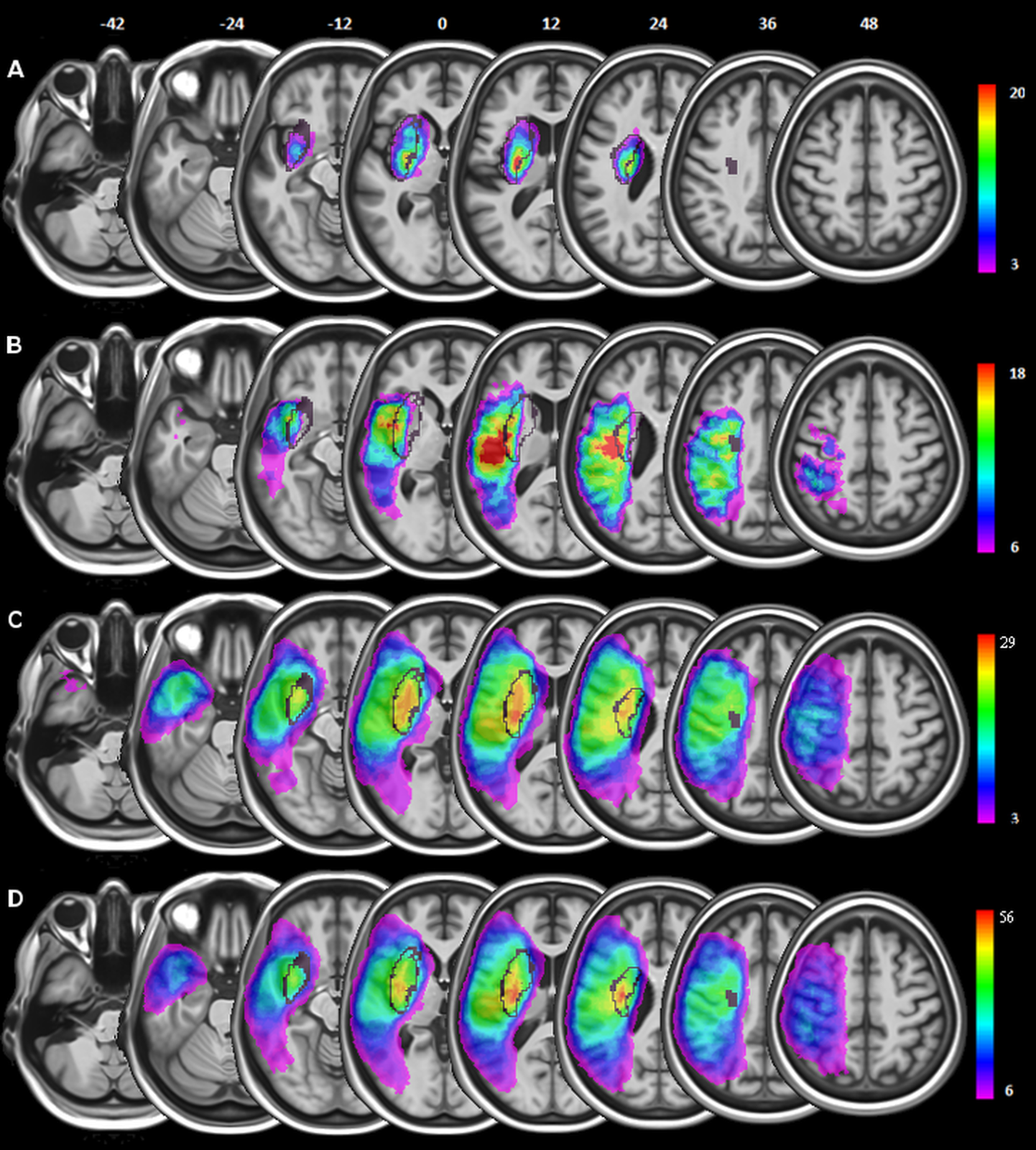


**Supplementary Figure S2**


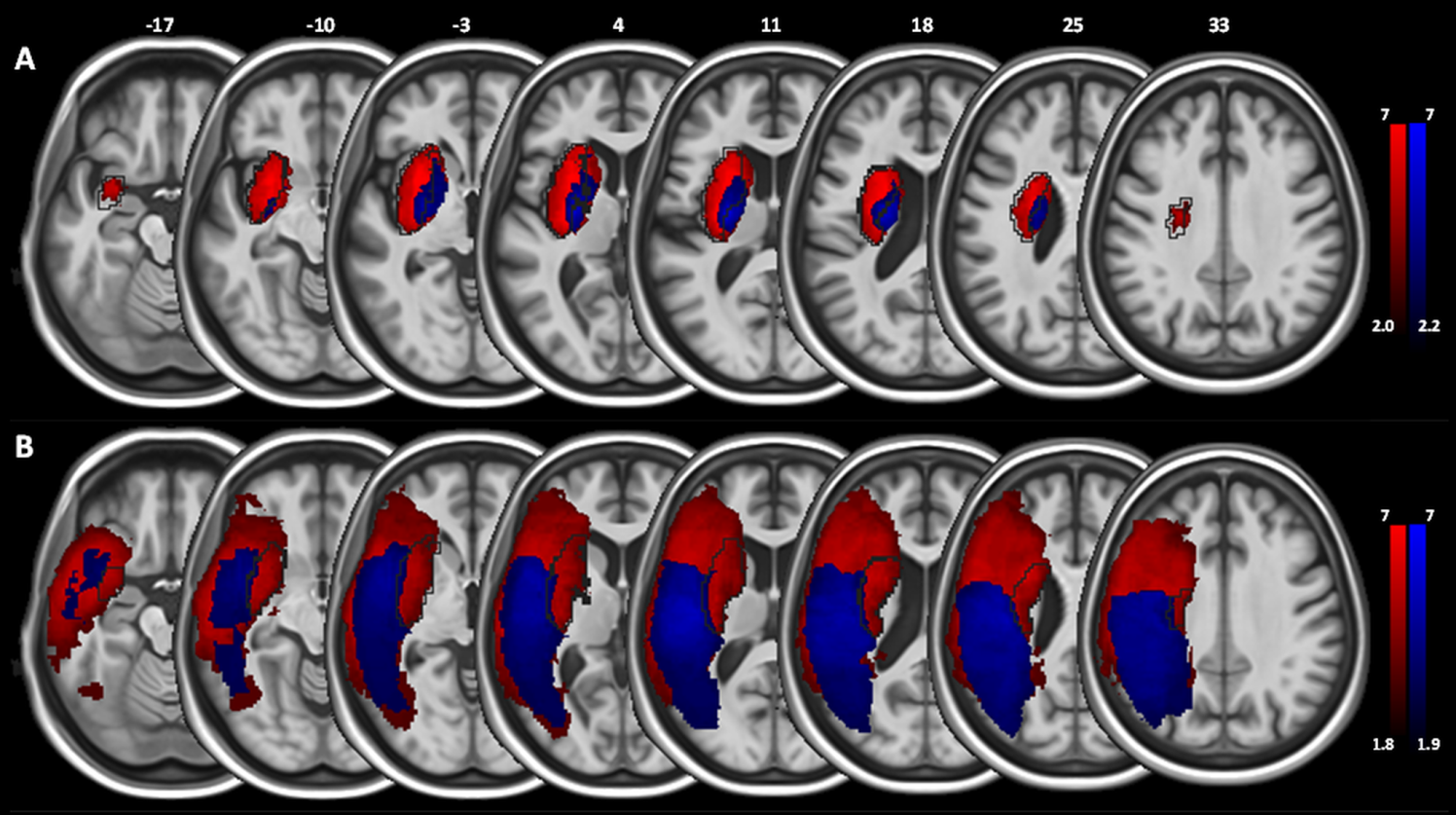


**Supplementary Figure S3**


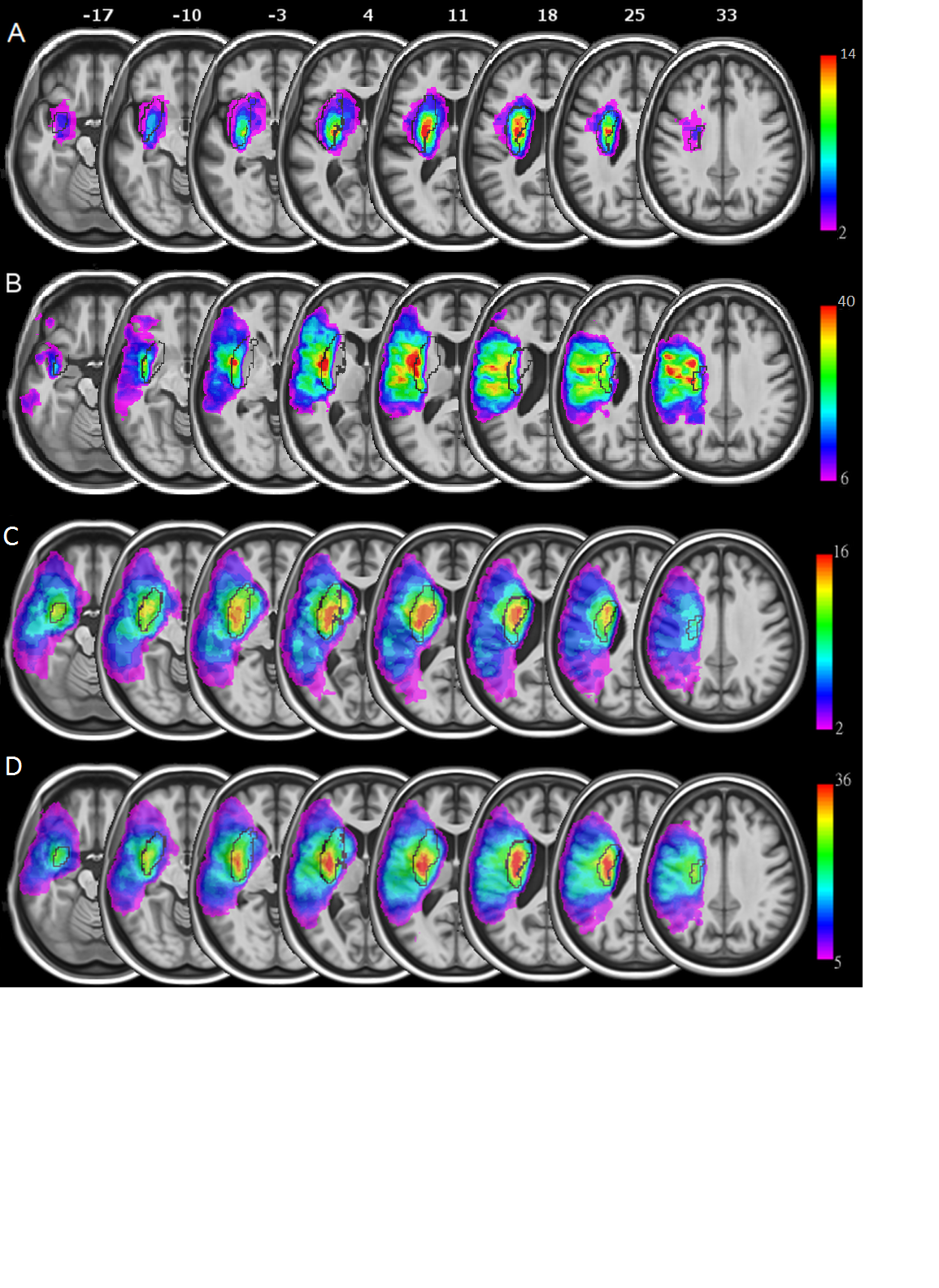


**Supplementary Figure S4**


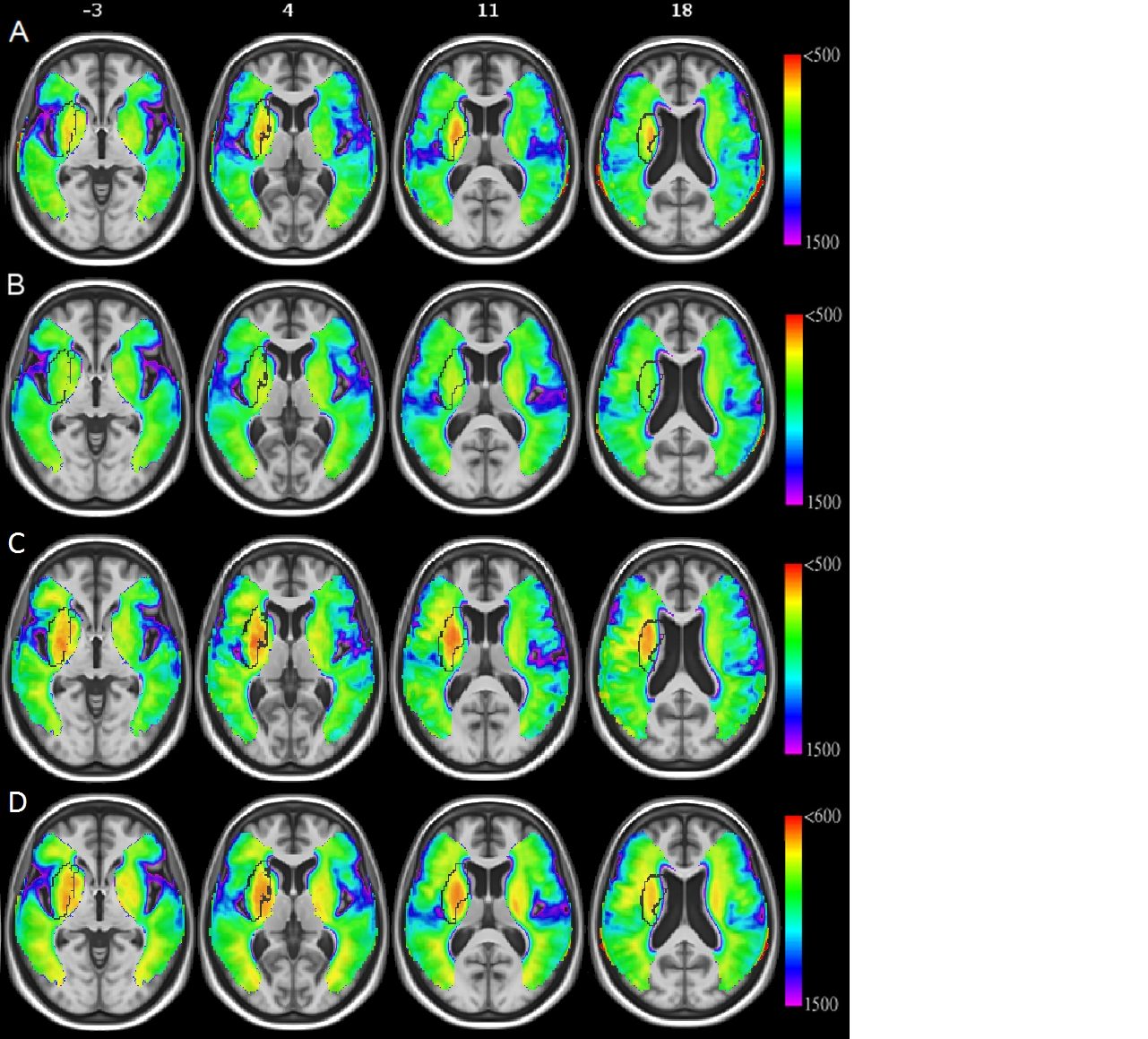

Supplement: Supplementary file 1 — Supplementary information. [file 41598_2020_58480_MOESM1_ESM.docx]
